# Reasons for participating in musical activities and their relationship with well-being during and before Covid-19

**DOI:** 10.1177/03057356221124034

**Published:** 2022-10-12

**Authors:** Luca Kiss, Karina J. Linnell

**Affiliations:** Department of Psychology, Goldsmiths, University of London, London, UK

**Keywords:** music, Covid-19, subjective well-being, eudaimonic well-being, identity and agency, mood regulation, relaxation and company

## Abstract

People tend to participate in musical activities—whether it is making or listening to music—for reasons that are related to basic psychological needs. This study explored whether the coronavirus pandemic (Covid-19) has changed the reasons for participating in musical activities and examined the relationship between these reasons and well-being during as well as before the pandemic. In total, 246 people (between 18 and 35 years) completed a survey during the pandemic, which contained questions relating to the reasons for participating in musical activities—namely the promotion of identity and agency, mood regulation, relaxation and company, enjoyment—and to subjective and eudaimonic well-being before and after the outbreak of the pandemic. Results showed that during the pandemic compared with before, people more often chose music to promote identity and agency, mood regulation, and relaxation and company. Two of the reasons that were invoked more often—namely identity and agency and mood regulation—positively predicted eudaimonic and subjective well-being, respectively, during the pandemic as well as before. Thus, people’s reasons for participating in musical activities during the pandemic compared with before changed in a direction consistent with increasing both eudaimonic and subjective well-being.

Music is universal and present in people’s lives all around the world. One of the reasons so many people participate in musical activities—whether it is making or listening to music—is the immense psychological benefits conferred by music, such as increased satisfaction with life (e.g., Weinberg & Joseph, 2017), mood regulation and positive emotions (e.g., Groarke & Hogan, 2016; Juslin & Sloboda, 2010; Laukka, 2007; Saarikallio, 2008; Swaminathan & Schellenberg, 2016; Västfjäll et al., 2012), social bonding (e.g., Groarke & Hogan, 2016; Keeler et al., 2015; Papinczak et al., 2015; Tarr et al., 2016), relaxation and reduced stress (e.g., Baltazar et al., 2019; de Witte et al., 2020; Groarke & Hogan, 2016; Laukka, 2007; Lynar et al., 2017), and identity and personal agency (Laukka, 2007; North & Hargreaves, 1999; Saarikallio et al., 2020). In fact, Laukka (2007) highlighted that people tend to listen to music for reasons that are related to basic psychological needs such as pleasure, mood regulation, relaxation, agency, identity, and belonging. These are all significant components of psychological well-being, where well-being can be defined by two components: *subjective* well-being including affect regulation (Diener et al., 2003; Scherer et al., 2004) and satisfaction with life (Diener et al., 1985), and *eudaimonic* well-being or the realisation of personal potential in the fulfillment of personal goals including environmental mastery, personal growth, autonomy, self-acceptance, and positive relations with others (Deci & Ryan, 2000; Laukka, 2007).

Although the positive relationship between listening to and making music and well-being has already been established (see, for example, Croom, 2015; Daykin et al., 2018, for reviews), studies focusing on this topic were mostly conducted during average life-situations and not during times of crisis such as the current pandemic. As a result of the coronavirus pandemic (Covid-19) caused by the SARS CoV-2 virus, regional lockdowns and restrictions on travel and social contact were introduced, available services and general income have reduced, and there were considerable changes in studying and working conditions. As a consequence of these restrictions, many people have experienced heightened levels of stress, uncertainty, and anxiety (for reviews, see Pedrosa et al., 2020; Vindegaard & Benros, 2020) and decreased well-being (e.g., Brooks et al., 2020; Gao et al., 2020; McGinty et al., 2020; O’Connor et al., 2021; Pierce et al., 2020; Robb et al., 2020; World Health Organization [WHO], 2020; Zacher & Rudolph, 2021). Given the positive effects of music on well-being, music could serve as an effective tool to cope with the negative effects of Covid-19 (Porshi, 2020).

The few studies exploring the possibility of music being a helpful tool during Covid-19 have indeed found that music listening can increase satisfaction with life (Krause et al., 2021) and that participating in musical activities such as listening, singing, dancing, or playing an instrument can help people relax, escape, raise their mood, and keep them company (Cabedo-Mas et al., 2021) and decrease worry and anxiety (Carlson et al., 2021). This study aimed to extend these recent findings and, based on Laukka’s (2007) pre-pandemic findings that certain reasons for music listening are strongly linked to well-being, directly explore how reasons for participating in musical activities—whether listening to or making music—are correlated with both subjective and eudaimonic well-being during the pandemic. By exploring how participants’ reported reasons for music participation and their relationship with well-being during the pandemic differed from their recollections of their reasons for music participation and well-being before the outbreak of the pandemic, collected during the pandemic, we aimed to shed more light on the nature of the more general relationship between participating in musical activities and well-being (e.g., Croom, 2015; Daykin et al., 2018) and specifically on why such participation increases well-being.

In sum, in this study, we first focused on whether reasons for participating in musical activities—specifically identity and agency, mood regulation, relaxation and company, and enjoyment, all as defined in Laukka (2007)—have changed after the outbreak of the pandemic compared with before. Second, we focused on whether these reasons are related to subjective (positive affect, satisfaction with life) and eudaimonic (environmental mastery, personal growth, autonomy, self-acceptance, positive relations with others) well-being during the pandemic and how if at all they are related differently than before.

## Method

### Design

The study was a cross-sectional survey study completed during the pandemic, which aimed to compare self-reported recollections of before the outbreak of the pandemic with current lived experiences after the outbreak of the pandemic. Self-report measures included music- and well-being-related variables related to the reasons for participating in musical activities (*identity and agency*, *mood regulation*, *relaxation and company*, *enjoyment*) and to subjective (*positive affect*, *satisfaction with life*) and eudaimonic (*environmental mastery*, *personal growth*, *autonomy*, *self-acceptance*, *positive relations with others*) well-being (see section “Materials”).

### Participants

Data were collected online with an opportunity sampling strategy: the survey was advertised on Facebook, SurveyCircle, and Goldsmiths’ Research Participation Database in exchange for credits for Psychology students. Anyone above the age of 18 could participate; there were no other inclusion criteria. There were 246 participants in total, 45 males and 201 females, mainly living in the United Kingdom. Given the difference in the proportions of biological sex, all analyses were completed not only on the whole dataset but also on the subset of females (201 participants). Because these analyses showed the same significant results, analyses on the full dataset only are included in the article.

This study focused on the young adult and adult population and included 185 participants between the ages of 18 and 25 years and 61 participants between the ages of 26 and 35 years. There were 153 participants whose highest education level was high school, 46 who held a bachelor’s degree, 32 who held a master’s degree, 12 who held a PhD, and 3 who held another form of professional degree. In all, 191 participants indicated that they were students, 70 employed, 12 self-employed, 7 out of work, 8 out of work because of Covid-19, and 2 homemakers (for this question only, participants were allowed to choose all the options that applied to them).

In addition to participants being asked about their demographic background, they were asked about their current situation regarding Covid-19. In total, 130 participants reported that they were going out for essentials but otherwise staying at home, 61 reported that they were going out to work but avoiding social contact with others, 28 reported that they were staying at home at all times, 19 reported that they did not engage in social distancing, and 8 reported that they were either quarantined or self-isolating. Out of the 246 participants, 21 had previously tested positive for Covid-19 and 15 lost a loved one due to Covid-19, with an additional 4 participants reporting that their loved one’s condition was critical.

**Figure 1.**
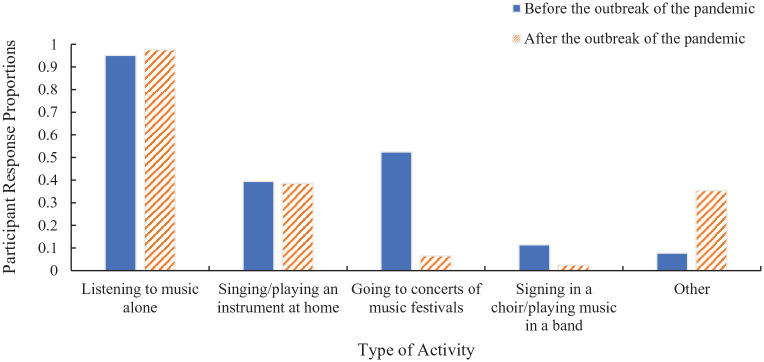
Participant Response Proportions for Each Musical Activity Before and After the Outbreak of the Pandemic. Responses in the “Other” Category Included Before the Outbreak of the Pandemic: Signing in School; Mixing/Writing Music; Signing with an Instructor; Going to Musicals; Listening to Music During Sports, Work, Driving, and with Friends at Social Gatherings. Responses in the “Other” Category Included After the Outbreak of the Pandemic: Watching Prerecorded or Live Online Music Videos; Singing/Playing Music with Others at Home or Online from Home; Mixing/Writing Music; Listening to Music During Sports, During Working from Home, and During Driving.

Furthermore, in addition to participants being asked about their situation regarding Covid-19, they were asked to retrospectively report the type of musical activities that they participated in before the outbreak of the pandemic and to report the activities that they were participating in after the outbreak of pandemic (Figure 1).

Participants who only listened to music compared with those who made music as well as listened to music did not differ in their reported well-being, as retrospectively reported before, or experienced after, the outbreak of the pandemic. Given this, the difference in the type of musical participation was not included in the main analyses reported in the “Results” section.

### Materials

To assess the effect of the pandemic, music- and well-being-related questions were presented to the participants twice—once referring to their recollection of memories from before the outbreak of the pandemic (e.g., “How often did you engage in musical activities for the following reasons before the outbreak of the pandemic?”) and once referring to their current lived experiences after the outbreak of the pandemic (e.g., “How often do you engage in musical activities now for the following reasons?”). Although the majority of the participants applied for participation credits and were thus Goldsmiths’ students based in the United Kingdom, the study was also advertised online with a view to allowing participants outside the United Kingdom to participate. Thus, the specific date for the outbreak of the pandemic was not specified to accommodate the different times when the pandemic broke out in the different countries and regions where respondents were living. It was made clear to participants at the beginning of the survey that the focus of the study was on change *since* the pandemic with the intention that they would report their experience during the pandemic and just before its outbreak.

#### Reasons for music participation

Questions were based on Laukka (2007) but were presented to the participants twice, once referring to before the outbreak of the pandemic and once referring to after. The questions referred to four types of reasons for music participation, specifically participating in musical activities for identity and agency, for mood regulation, for relaxation and company, and for enjoyment. Participants were presented with a large list of potential strategies which after data collection were grouped into four main categories (i.e., *identity and agency*, *mood regulation*, *relaxation and company*, *enjoyment*) based on Laukka’s (2007) factor analysis on the items. To assess how often participants engaged in musical activities for identity and agency, they were asked to signal to what extent each of the following statements applied to them on a scale from 1 *(never)* to 6 *(always)*: “It strengthens my self-image,” “To express my personality,” “To shield out the world around me,” “To strengthen self-esteem,” “It makes me feel competent,” “To gain control of sound in my surroundings.” Similarly, to assess music participation for mood regulation, they were presented with the following statements: “It evokes memories,” “Music induces emotions,” “To enhance positive moods,” “To vent emotions,” “To reflect on my life,” “To feel akin to others.” To assess music participation for relaxation and company, they were presented with the following statements: “To have something in the background,” “To forget about the present,” “To stir up energy,” “To relax and calm down,” “To reduce feelings of loneliness.” Finally, to assess music participation for enjoyment, they were presented with the following statements: “For entertainment,” “It gives me pleasure,” “Interest in music itself,” “It is beautiful.”

#### Well-being

Subjective well-being was measured by both its affective and cognitive components. The affective component, positive affect, was measured with the WHO-5 Well-being Index (Regional Office for Europe, World Health Organization, 1998). This short questionnaire has been widely used to assess subjective well-being (Topp et al., 2015). It includes five positively phrased statements related to positive affect. In the questionnaire, participants are asked to rate how much of the time they felt each subjective state (e.g., cheerful, calm, active, fresh, interested) during the past 2 weeks (0 = *at no time* through to 5 = *all of the time*). For the purpose of this study, they were also asked to indicate their well-being before the pandemic. The WHO-5 index has been found to have high construct validity for measuring subjective well-being (Topp et al., 2015).

The cognitive component of subjective well-being, satisfaction with life, was measured with the Satisfaction With Life Scale (Diener et al., 1985; Pavot & Diener, 1993). This scale contains five short statements to measure participants’ satisfaction with their life. Participants were asked to indicate how much they agreed or disagreed with the statements (1 = *strongly disagree* through to 7 = *strongly agree*). Pavot and Diener (1993) showed high content validity and reliability of the scale, and Laukka (2007) found high internal consistency.

Similarly to Laukka (2007), eudaimonic well-being was measured with Ryff’s (1989) Scales of Psychological Well-Being, in which participants are asked to indicate on a 6-point scale how much they agree or disagree (1 = *strongly disagree* through to 6 = *strongly agree*) with a set of statements. This measure originally contained six 3-item sub-scales measuring self-acceptance (positive attitudes toward oneself), positive relations with others (the ability to achieve close relationships with others), autonomy (qualities of self-determination, independence, and self-regulation of behavior), environmental mastery (ability to engage in and manage tasks in one’s surrounding), personal growth (one’s personal development and commitment to realizing potential to grow as a person), and purpose in life (Clarke et al., 2001; Ryff & Keyes, 1995). Although previous research has shown acceptable validity (e.g., Clarke et al., 2001) and moderate internal consistency (Laukka, 2007; Waterman et al., 2010) of the sub-scales, purpose in life showed very low internal alpha levels (Laukka, 2007). Thus, similarly to Laukka (2007), the present survey excluded the purpose in life sub-scale and contained 14 items in total. Consequently, all dimensions were assessed with three items, apart from environmental mastery which was assessed with only two items due to technical issues.

### Procedure

The survey received ethical approval on June 15, 2020, from Goldsmiths, University of London. Data for this study were collected between June and November 2020. Analyses on the effect of time of completion of the survey showed no significant results. Completion of the survey took approximately 20–30 min.

After reading the information sheet, General Data Protection Regulation, and signing the consent form, participants completed the questions asking about their demographic background, current situation regarding Covid-19, their reasons for participating in musical activities, and subjective and eudaimonic well-being. At the end, participants were presented with the debrief sheet and university students were asked to leave their username to receive the credits.

## Results

The aims of this study were to explore how Covid-19 has affected the reasons for participating in musical activities and whether the reasons for music participation can predict subjective and eudaimonic well-being during and before Covid-19.

### Reasons for music participation

**Figure 2.**
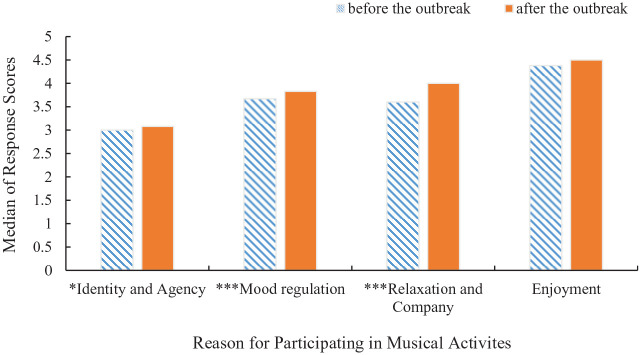
Median of Response Scores for Reasons for Music Participation as a Function of the Outbreak of the Pandemic (Before/After). The Relevant Question in the Survey was “How Often do you Engage in Musical Activities Because of the Following Reasons?” And Answers were Rated on a 6-Point Scale (1 = *Never* Through to 6 = *Always*). *p < .01, ***p < .001.

First, we explored whether participants’ reported reasons for participating in musical activities during the pandemic differed from their recollected reasons for participating before the pandemic. Wilcoxon signed-rank tests showed that there was a significant difference between scores given for reasons for music participation before and after the outbreak of the pandemic, specifically for the reasons of identity and agency, *z* = −2.44, *p* = .015, *r* = .156, mood regulation, *z* = −3.56, *p* < .001, *r* = .227, and relaxation and company, *z* = −5.78, *p* < .001, *r* = .369. Specifically, participants reported participating in musical activities *more* often for mood regulation, for relaxation and company, and for identity and agency after the outbreak of the pandemic than before (see Figure 2).

### The relationship between reasons for music participation and well-being

To directly investigate whether reasons for music participation are linked to well-being, multiple linear regression analyses were conducted for participants’ recollections of before the outbreak of the pandemic and for their current lived experiences after the outbreak of the pandemic: similarly to Laukka (2007), the regression analyses were set up to explore whether reasons for music participation (identity and agency, mood regulation, relaxation and company, enjoyment) can predict aspects of subjective (positive affect, satisfaction with life) and eudaimonic (environmental mastery, personal growth, autonomy, self-acceptance, positive relations with others) well-being. For each well-being dependent variable, a separate regression model was calculated.

Results showed similar significant relationships between participants’ recollections of their reasons for music participation and well-being before the outbreak of the pandemic compared with between their reported reasons for music participation and well-being after the outbreak (see Table 1). Although Fisher’s tests showed no significant change in the strength of the relationships that existed before and after the outbreak of the pandemic, there were more significant relationships after the outbreak: After the outbreak of the pandemic, participating in musical activities for identity and agency was a unique positive predictor of aspects of eudaimonic well-being (personal growth, autonomy, positive relations with others) and participating in musical activities for mood regulation was a unique positive predictor of subjective well-being (positive affect and satisfaction with life) over and above the other predictor variables. While the reason of mood regulation was negatively linked to autonomy before the outbreak of the pandemic, this relationship disappeared after the outbreak of the pandemic. Participating in musical activities for the reason of relaxation and company was the only reason that did not predict any of the well-being variables, whereas participating in musical activities for enjoyment was a unique negative predictor of all well-being variables.

**Table 1.** Reasons for music participation as predictors of well-being before and after the outbreak of the pandemic: squared multiple correlations (*R*^2^) with significance of the overall model (*), beta weights (β), *t* values (degrees of freedom), and squared part correlations (sr^2^) of the models (separate model for each well-being variable).

Before the outbreak of the pandemic						After the outbreak of the pandemic					
		Well-being variables (*R*^2^)		Reasons for music participation				Well-being variables (*R*^2^)		Reasons for music participation	
		Identity and agency	Mood regulation	Relaxation and company	Enjoyment			Identity and agency	Mood regulation	Relaxation and company	Enjoyment
Subjective well-being	Positive affect (.07**)	**ns**	**ns**	ns	**[β = −5.25, τ(238) = −3.17, σρ** ^2^ = .04**]	Subjective well-being	Positive affect (.10***)	ns	β = 4.65, τ(239) = 1.98, σρ^2^ = .01*	ns	β = −8.19, τ(239) = −4.97, σρ^2^ = .10***
	Satisfaction with life (.04*)	ns	β= 1.70, τ(239) = 2.11, σρ^2^ = .02*	ns	β = −1.70, τ(239) = −3.00, σρ^2^ =.03**		Satisfaction with life (.09***)	ns	β = 2.10, τ(240) = 2.71, σρ^2^ = .03**	ns	β = −2.03, τ(240) = −3.76, σρ^2^ = .05***
Eudaimonic well-being	Environmental mastery (.09***)	ns	ns	**ns**	β = −.83, τ(239) = −4.39, σρ^2^ = .07***	Eudaimonic well-being	Environmental mastery (.08***)	ns	ns	ns	β = −0.91, τ(240) = −4.35, σρ^2^ = .07***
	Personal growth (.08**)	β = .70, τ(239) = 3.43, σρ^2^ = .04**	**ns**	ns	β = −.69, τ(239) = −3.12, σρ^2^ = .04**		Personal growth (.09***)	β = .74, τ(240) = 3.65, σρ^2^ = .05***	ns	ns	β = −.66, τ(240) = −3.13, σρ^2^ = .04**
	Autonomy (.06**)	ns	β = −.83, τ(239) = −2.25, σρ^2^ = .02*	ns	β = −.59, τ(239) = −3.21, σρ^2^ = .04*		Autonomy (.07**)	β = .71, τ(240) = 2.81, σρ^2^ = .03**	ns	ns	β = −1.00, τ(240) = −3.82, σρ^2^ = .06***
	Self-acceptance (.05*)	**ns**	ns	ns	β = −.93, *τ*(239) = −3.91, σρ^2^ = .06***		Self-acceptance (.06**)	ns	ns	ns	β = −1.12, τ(240) = −3.91, σρ^2^ = .06***
	Positive relations with others (.09***)	β = .68, τ(239) = 2.77, σρ^2^ = .03**	ns	ns	β = −1.20, τ(239) = −3.81, σρ^2^ = .06***		Positive relations with others (.07**)	β = .71, τ(240) = 2.59, σρ^2^ = .03*	ns	ns	β = −.86, τ(240) = −3.03, σρ^2^ = .04**

ns: non-significant.

Bold represents significant relationships reported in Laukka (2007), and square brackets represent significant relationships reported in Laukka (2007) but in the opposite direction to the current results.

* *p*<.05. **p<.01. ***p<.001.

## Discussion

The present study explored the effects of Covid-19 on the reasons for participating in musical activities and their relationship with subjective and eudaimonic well-being. The results showed that participants reported participating in musical activities more often for identity and agency, mood regulation, and relaxation and company, after the outbreak of the pandemic than they retrospectively recalled doing so before. These results extend findings by Cabedo-Mas et al. (2021) and Carlson et al. (2021) showing that during the pandemic the majority of participants reported that music improves their mood, helps them relax, and keeps them company.

An explanation for the present finding on mood regulation and relaxation could be that, because Covid-19 increased people’s negative arousal due to the stress and anxiety it caused (e.g., Pedrosa et al., 2020; Vindegaard & Benros, 2020), people might have turned to music more often to reduce and regulate their arousal. Past research supports this explanation and shows that music can regulate arousal (e.g., Schäfer et al., 2013) and help people become more relaxed (e.g., Lynar et al., 2017; Pelletier, 2004). Also, people might have turned to music for company more often as a result of the increase in loneliness and isolation due to Covid-19 (e.g., Brooks et al., 2020; McGinty et al., 2020). Furthermore, they might have turned to music for identity and agency more often as a result of the increase in uncertainty and decrease in the control they had over their surroundings (Rettie & Daniels, 2021). In sum, the increased participation in musical activities to feel a sense of identity and agency, to regulate mood, to feel relaxed, and to have a sense of company might be an effective tool to cope with the negative effects of Covid-19.

Indeed, as predicted, two of the reasons that increased after the outbreak of the pandemic were positively linked to well-being: After the outbreak of the pandemic, participating in musical activities for identity and agency significantly predicted aspects of eudaimonic well-being (personal growth, autonomy, positive relations with others), and participating in musical activities for mood regulation significantly predicted subjective well-being (positive affect and satisfaction with life). These results were similar when recollections of the period before the outbreak of the pandemic were analyzed but relationships were less significant. Given the present finding that participants reported using music more often for identity and agency and mood regulation after the outbreak of the pandemic, and that these reasons are positively linked to eudaimonic and subjective well-being, respectively, it can be suggested that during the pandemic—and by implication in times of crisis—people more often turn to music for its psychological benefits, which in-turn helps them increase their well-being. However, given the correlational nature of the design, the causality of the relationships cannot be properly addressed; it cannot be ruled out that, instead of the reasons for music participation being the driver of changes in well-being, well-being is driving changes in the reasons, such that higher well-being leads to an increase in the frequency of participation for mood regulation and for identity and agency.

The positive link found in this study during the pandemic between well-being and reasons for participating in musical activities, namely identity and agency and mood regulation, extends the few studies focusing on Covid-19 and showing positive results on well-being of playing or listening to music: Participating in musical activities enhanced satisfaction with life (Krause et al., 2021); listening to carefully curated music playlists enhanced emotional well-being, valence, and sense of meaning (Sice et al., 2020); and receptive music therapy enhanced emotional well-being and reduced stress during Covid-19 (Giordano et al., 2020). It also extends research *before* the pandemic on music listening in general (e.g., Groarke & Hogan, 2016; Morinville et al., 2013; Papinczak et al., 2015; Saarikallio, 2011) and on reasons for music listening in the elderly population in particular (e.g., Laukka, 2007).

While two of the three reasons for music participation that increased during the pandemic, namely identity and agency and mood regulation, were linked to well-being, the third reason, namely relaxation and company, was not linked to any of the well-being variables before or after the outbreak of the pandemic. The fourth reason for music participation, namely enjoyment, was alone in not increasing during the pandemic and indeed in having a negative relationship with both subjective and eudaimonic well-being, and then both before and after the outbreak of the pandemic. The direction of this relationship was not compatible with research by Laukka (2007) showing a positive, albeit weak, correlation between well-being and participating in musical activities—specifically listening—for enjoyment. Nonetheless, it is unlikely that the current negative relationship found here is an anomaly given the strong relationship across all well-being variables both before and after the outbreak of the pandemic. One is left to speculate about what is the direction of the causality: If one assumes that it is the reason for music participation that drives the well-being, one would have to argue that the more one uses music for enjoyment, the more well-being reduces. On the other hand, it might be that the causality is in the opposite direction such that those whose well-being is low are more inclined to participate in musical activities for enjoyment.

The present findings emphasise the urgency of resolving the question around the direction of the causality between reasons for music participation and well-being (see also Laukka, 2007). Future research should therefore more closely investigate potential factors that might mediate the relationship between reasons for music participation and well-being. This could then help inform why certain reasons might be related to well-being differently than others. For example, exploring the specific types of music (in terms of, for example, tempo, genre, valence) people choose for the different reasons could help to resolve the nature of the relationship between reasons for music participation and well-being.

One important limitation of this exploratory study is that participants were not given an explicit timeframe before the pandemic over which to report their recollected reasons for music participation and well-being before the pandemic. Nonetheless, it was made explicit to participants that the focus was on change *since* the pandemic, with the intention that participants would report their experience just before the outbreak of the pandemic. A second limitation is that reports relating to before the outbreak of the pandemic could have been limited by recall errors, resulting in either under- or over-reporting of experience (Kjellsson et al., 2014). Nevertheless, it is reassuring that not all the reasons for music participation increased during the pandemic and that the relationships between reasons for music participation and well-being were similar before the pandemic and during it.

## Conclusion

Music is universal and has many psychological benefits, including improving our well-being. The present survey study aimed to explore how the reasons for participating in musical activities have changed since the pandemic and whether these reasons are significant predictors of subjective and eudaimonic well-being during and before Covid-19. The results showed that after the outbreak of Covid-19, people participated in musical activities more often for reasons related to feeling a sense of identity and agency, regulating their mood, and feeling relaxed and in company. Importantly, some of the increased reasons, namely music participation for identity and agency and for mood regulation, could positively predict well-being during the pandemic. Together, these findings extend previous research by showing that reasons for music participation are significantly linked to well-being not only before but also during the pandemic and suggest that those who use music more for identity and agency and mood regulation during the pandemic show, respectively, greater eudaimonic and subjective well-being.

## References

[bibr1-03057356221124034] Baltazar M. Västfjäll D. Asutay E. Koppel L. Saarikallio S. (2019). Is it me or the music? Stress reduction and the role of regulation strategies and music. Musicae Sciantiae, 2, 1–16. 10.1177/2059204319844161

[bibr2-03057356221124034] Brooks S. K. Webster R. K. Smith L. E. Woodland L. Wessely S. Greenberg N. Rubin G. J. (2020). The psychological impact of quarantine and how to reduce it: Rapid review of the evidence. The Lancet, 395(10227), 912–920. 10.1016/S0140-6736(20)30460-8 PMC7158942 32112714

[bibr3-03057356221124034] Cabedo-Mas A. Arriaga-Sanz C. Moliner-Miravet L. (2021). Uses and perceptions of music in times of COVID-19: A Spanish Population Survey. Frontiers in Psychology, 11, Article 606180. 10.3389/fpsyg.2020.606180 PMC7835488 33510681

[bibr4-03057356221124034] Carlson E. Wilson J. Baltazar M. Duman D. Peltola H. R. Toiviainen P. Saarikallio S. (2021). The role of music in everyday life during the first wave of the Coronavirus pandemic: A mixed-methods exploratory study. Frontiers in Psychology, 12, Article 647756. 10.3389/fpsyg.2021.647756 PMC8129180 34017286

[bibr5-03057356221124034] Clarke P. J. Marshall V. W. Ryff C. D. Wheaton B. (2001). Measuring psychological well-being in the Canadian Study of Health and Aging. International Psychogeriatrics, 13(1), 79–90. 10.1017/s1041610202008013 11892978

[bibr6-03057356221124034] Croom A. M. (2015). Music practice and participation for psychological well-being: A review of how music influences positive emotion, engagement, relationships, meaning, and accomplishment. Musicae Scientiae, 19(1), 44–64. 10.1177/1029864914561709

[bibr7-03057356221124034] Daykin N. Mansfield L. Meads C. Julier G. Tomlinson A. Payne A. Grigsby D. L. Lane J. D’Innocenzo G. Burnett A. Kay T. Dolan P. Testoni S. Victor C. (2018). What works for wellbeing? A systematic review of wellbeing outcomes for music and singing in adults. Perspectives on Public Health, 138(1), 39–46. 10.1177/1757913917740391 PMC5753835 29130840

[bibr8-03057356221124034] Deci E. L. Ryan R. M. (2000). The “what” and “why” of goal pursuits: Human needs and the self-determination of behavior. Psychological Inquiry, 11(4), 227–268. 10.1207/S15327965PLI1104_01

[bibr9-03057356221124034] de Witte M. Spruit A. van Hooren S. Moonen X. Stams G. J . (2020). Effects of music interventions on stress-related outcomes: A systematic review and two meta-analyses. Health Psychology Review, 14, 294–324. 10.1080/17437199.2019 31167611

[bibr10-03057356221124034] Diener E. Emmons R. A. Larsen R. J. Griffin S. (1985). The Satisfaction With Life Scale. Journal of Personality Assessment, 49(1), 71–75. 10.1207/s15327752jpa4901_13 16367493

[bibr11-03057356221124034] Diener E. Oishi S. Lucas R. E. (2003). Personality, culture and subjective well-being: Emotional and cognitive evaluations of life. Annual Review of Psychology, 54, 403–424. 10.1146/annurev.psych.54.101601.145056 12172000

[bibr12-03057356221124034] Gao J. Zheng P. Jia Y. Chen H. Mao Y. Chen S. Wang Y. Fu H. Dai J. (2020). Mental health problems and social media exposure during COVID-19 outbreak. PLOS ONE, 15(4), Article e0231924. 10.1371/journal.pone.0231924 PMC7162477 32298385

[bibr13-03057356221124034] Giordano F. Scarlata E. Baroni M. Gentile E. Puntillo F. Brienza N. Gesualdo L. (2020). Receptive music therapy to reduce stress and improve wellbeing in Italian clinical staff involved in COVID-19 pandemic: A preliminary study. The Arts in Psychotherapy, 70, Article 101688. 10.1016/j.aip.2020.101688 PMC7361107 32834302

[bibr14-03057356221124034] Groarke J. M. Hogan M. J. (2016). Enhancing wellbeing: An emerging model of the adaptive functions of music listening. Psychology of Music, 44(4), 769–791. 10.1177/0305735615591844

[bibr15-03057356221124034] Juslin P. N. Sloboda J. A. (Eds.). (2010). Handbook of music and emotion: Theory, research, applications. Oxford University Press.

[bibr16-03057356221124034] Keeler J. R. Roth E. A. Neuser B. L. Spitsbergen J. M. Waters D. J. M. Vianney J. M. (2015). The neurochemistry and social flow of singing: Bonding and oxytocin. Frontiers in Human Neuroscience, 9, Article 518. 10.3389/fnhum.2015.00518 PMC4585277 26441614

[bibr17-03057356221124034] Kjellsson G. Clarke P. Gerdtham U. G. (2014). Forgetting to remember or remembering to forget: A study of the recall period length in health care survey questions. Journal of Health Economics, 35, 34–46. 10.1016/j.jhealeco.2014.01.007 24595066

[bibr18-03057356221124034] Krause A. E. Dimmock J. Rebar A. L. Jackson B. (2021). Music listening predicted improved life satisfaction in university students during early stages of the Covid-19 pandemic. Frontiers in Psychology, 11, Article 4022. 10.3389/fpsyg.2020.631033 PMC7855032 33551940

[bibr19-03057356221124034] Laukka P. (2007). Uses of music and psychological well-being among the elderly. Journal of Happiness Studies: An Interdisciplinary Forum on Subjective Well-Being, 8(2), 215–241. 10.1007/s10902-006-9024-3

[bibr20-03057356221124034] Lynar E. Cvejic E. Schubert E. Vollmer-Conna U. (2017). The joy of heartfelt music: An examination of emotional and physiological responses. International Journal of Psychophysiology, 120, 118–125. 10.1016/j.ijpsycho.2017.07.012 28757232

[bibr21-03057356221124034] McGinty E. E. Presskreischer R. Han H. Barry C. L. (2020). Psychological distress and loneliness reported by US adults in 2018 and April 2020. Journal of the American Medical Association, 324(1), 93–94. 10.1001/jama.2020.9740 32492088 PMC7270868

[bibr22-03057356221124034] Morinville A. Miranda D. Gaudreau P. (2013). Music listening motivation is associated with global happiness in Canadian late adolescents. Psychology of Aesthetics, Creativity, and the Arts, 7(4), 384–390. 10.1037/a0034495

[bibr23-03057356221124034] North A. C. Hargreaves D. J. (1999). Music and adolescent identity. Music Education Research, 1, 75–92. 10.1080/1461380990010107

[bibr24-03057356221124034] O’Connor R. Wetherall K. Cleare S. McClelland H. Melson A. Niedzwiedz C. O’Carroll R. E. O’Connor D. B. Platt S. Scowcroft E. Watson B. Zortea T. Ferguson E. Robb K. (2021). Mental health and well-being during the COVID-19 pandemic: Longitudinal analyses of adults in the UK COVID-19 Mental Health & Wellbeing Study. The British Journal of Psychiatry, 218(6), 326–333. 10.1192/bjp.2020.212 33081860 PMC7684009

[bibr25-03057356221124034] Papinczak Z. E. Dingle G. A. Stoyanov S. R. Hides L. Zelenko O. (2015). Young people’s uses of music for well-being. Journal of Youth Studies, 18(9), 1119–1134. 10.1080/13676261.2015.1020935

[bibr26-03057356221124034] Pavot W. Diener E. (1993). Review of the Satisfaction With Life Scale. Psychological Assessment, 5(2), 164–172. 10.1037/1040-3590.5.2.164

[bibr27-03057356221124034] Pedrosa A. L. Bitencourt L. Froes A. C. F. Cazumba M. L. B. Campos R. G. B. de Brito S. Silva A. (2020). Emotional, behavioral, and psychological impact of the COVID-19 pandemic. Frontiers in Psychology, 11(18), Article 566212. 10.3389/fpsyg.2020.566212 PMC7561666 33117234

[bibr28-03057356221124034] Pelletier C. L. (2004). The effect of music on decreasing arousal due to stress: A meta-analysis. Journal of Music Therapy, 41(3), 192–214. 10.1093/jmt/41.3.192 15327345

[bibr29-03057356221124034] Pierce M. Hope H. Ford T. Hatch S. Hotopf M. John A. Kontopantelis E. Webb R. Wessely S. McManus S. Abel K. M. (2020). Mental health before and during the COVID-19 pandemic: A longitudinal probability sample survey of the UK population. Lancet Psychiatry, 7(10), 883–892. 10.1016/s2215-0366(20)30308-4 32707037 PMC7373389

[bibr30-03057356221124034] Porshi J. M. (2020). Music reliefs stress & anxiety during COVID 19 pandemic. Asian Research Journal of Arts & Social Sciences, 11(4), 38–42. 10.9734/arjass/2020/v11i430178

[bibr31-03057356221124034] Rettie H. Daniels J. (2021). Coping and tolerance of uncertainty: Predictors and mediators of mental health during the COVID-19 pandemic. American Psychologist, 76(3), 427–437. 10.1037/amp0000710 32744841

[bibr32-03057356221124034] Robb C. E. de Jager C. A. Ahmadi-Abhari S. Giannakopoulou P. Udeh-Momoh C. McKeand J. Price G. Car J. Majeed A. Ward H. Middleton L. (2020). Associations of social isolation with anxiety and depression during the early COVID-19 pandemic: A survey of older adults in London, UK. Frontiers in Psychiatry, 11, Article 591120. 10.3389/fpsyt.2020.591120 PMC7566017 33132942

[bibr33-03057356221124034] Ryff C. D. (1989). Happiness is everything, or is it? Explorations of the meaning of psychological well-being. Journal of Personality and Social Psychology, 57(6), 1069–1081. 10.1037/0022-3514.57.6.1069

[bibr34-03057356221124034] Ryff C. D. Keyes C. L. (1995). The structure of psychological well-being revisited. Journal of Personality and Social Psychology, 69(4), 719–727. 10.1037/0022-3514.69.4.719 7473027

[bibr35-03057356221124034] Saarikallio S. (2008). Music in mood regulation: Initial scale development. Musicae Scientiae, 12, 291–309. 10.1177/102986490801200206

[bibr36-03057356221124034] Saarikallio S. (2011). Music as emotional self-regulation throughout adulthood. Psychology of Music, 39(3), 307–327. 10.1177/0305735610374894

[bibr37-03057356221124034] Saarikallio S. Randall W. M. Baltazar M. (2020). Music listening for supporting adolescents’ sense of agency in daily life. Frontiers of Psychology, 10, Article 2911. 10.3389/fpsyg.2019.02911 PMC6960221 32010014

[bibr38-03057356221124034] Schäfer T. Sedlmeier P. Städtler C. Huron D. (2013). The psychological functions of music listening. Frontiers in Psychology, 4, Article 511. 10.3389/fpsyg.2013.00511 PMC3741536 23964257

[bibr39-03057356221124034] Scherer K. R. Wranik T. Sangsue J. Tran V. Scherer U. (2004). Emotions in everyday life: Probability of occurrence, risk factors, appraisal and reaction patterns. Social Science Information, 43(7), 499–570. https://doi.org/10.1177%2F0539018404047701

[bibr40-03057356221124034] Sice P. Elvin G. Riachy C. Shang Y. Ogwu S. Zink C. (2020). Online screening of X-System music playlists using an integrative wellbeing model informed by the theory of autopoiesis. IEEE Access, 8, 182307–182319. 10.1109/ACCESS.2020.3029142

[bibr41-03057356221124034] Swaminathan S. Schellenberg E. G. (2016). Music training. In Strobach T. Karbach J. (Eds.), Cognitive training: An overview of features and applications (pp. 137–144). Springer. 10.1007/978-3-319-42662-4_13

[bibr42-03057356221124034] Tarr B. Launay J. Dunbar R. I. M. (2016). Silent disco: Dancing in synchrony leads to elevated pain thresholds and social closeness. Evolution and Human Behavior, 37, 343–349. 10.1016/j.evolhumbehav.2016.02.004 27540276 PMC4985033

[bibr43-03057356221124034] Topp C. W. Østergaard S. D. Søndergaard S. Bech P. (2015). The WHO-5 Well-Being Index: A systematic review of the literature. Psychotherapy and Psychosomatics, 84(3), 167–176. 10.1159/000376585 25831962

[bibr44-03057356221124034] Västfjäll D. Juslin P. N. Hartig T. (2012). Music, subjective wellbeing, and health: The role of everyday emotions. In MacDonald R. A. R. Kreutz G. Mitchell L. (Eds.), Music, health, and wellbeing (pp. 405–423). Oxford University Press. 10.1093/acprof:oso/9780199586974.003.0027

[bibr45-03057356221124034] Vindegaard N. Benros M. E. (2020). COVID-19 pandemic and mental health consequences: Systematic review of the current evidence. Brain Behavior and Immunity, 89, 531–542. 10.1016/j.bbi.2020.05.048 32485289 PMC7260522

[bibr46-03057356221124034] Waterman A. S. Schwartz S. J. Zamboanga B. L. Ravert R. D. Williams M. K. Agocha V. B. Kim S. Y. Donnellan M. B. (2010). The questionnaire for eudaimonic well-being: Psychometric properties, demographic comparisons, and evidence of validity. The Journal of Positive Psychology, 5(1), 41–61. 10.1080/17439760903435208 34326891 PMC8317967

[bibr47-03057356221124034] Weinberg M. K. Joseph D. (2017). If you’re happy and you know it: Music engagement and subjective wellbeing. Psychology of Music, 45(2), 257–267. 10.1177/0305735616659552

[bibr48-03057356221124034] World Health Organization. (2020). Substantial investment needed to avert mental health crisis. https://www.who.int/news/item/14-05-2020-substantial-investment-needed-to-avert-mental-health-crisis

[bibr49-03057356221124034] Regional Office for Europe, World Health Organization. (1998). Well-being measures in primary health care (The DepCare Project—Health for all, Target 12, E.60246). https://www.euro.who.int/__data/assets/pdf_file/0016/130750/E60246.pdf

[bibr50-03057356221124034] Zacher H. Rudolph C. W. (2021). Individual differences and changes in subjective wellbeing during the early stages of the COVID-19 pandemic. American Psychologist, 76(1), 50–62. 10.1037/amp0000702 32700938

